# Supplementary material for: Dynamic Interplay of Online Risk and Resilience in Adolescence (DIORA): a protocol for a 12-month prospective observational study testing the associations among digital activity, affective and cognitive reactions and depression symptoms in a community sample of UK adolescents
Source: BMJ Open. 2024 Sep 30;14(9):e085061. doi: 10.1136/bmjopen-2024-085061 (PMC11448176; doi:10.1136/bmjopen-2024-085061)
Supplement: Supplementary data [file bmjopen-14-9-s001.pdf]

Table 1. DIORA constructs, outcome, measures and schedule of assessments.

General construct	Outcome	Measure	T1	T2	T3
Demographic information		Adolescent age, biological sex, gender identity, ethnicity	A, P		
		Family affluence, eligibility for free school meals	A		
		Parental ethnicity, parental education, and employment status, main earner occupation.	P		
Adolescent psychopathology	Depression symptoms (primary outcome at T3)	Revised Child Anxiety and Depression Scale 25–Youth Version (RCADS-25; Ebesutani et al., 2017)	A, P	A, P	A, P
	Anxiety symptoms	Anxiety subscale of the RCADS-25.	A, P	A, P	A, P
	ADHD symptoms and conduct problems	Respective subscales of The Strengths and Difficulties Questionnaire (SDQ; Goodman, 1997).	A		A
Adolescent wellbeing	Wellbeing	Warwick-Edinburgh Mental Wellbeing Scale (WEMWBS; Tennant et al., 2007)	A, P	A, P	A, P
Digital activity and screen time	Amount of time spent online	Screen time measure	A	A	A
	Digital activity	*My Online Activity* subscale of the Digital Activity and Feelings Inventory (DAFI)	A	A	A
	Emotional reactions induced by digital activity	*My Feelings Online* subscale of the DAFI	A	A	A
	Impact of digital activity on daily functioning and concerns about the impact of digital activity on mental health and relationships	**My Life Online** (MYLO) questionnaire. Section one (9 items), asks adolescents about how much their life was impacted by their digital activity (e.g., “I missed meals”, “I had difficulties at school/college/university”) rated on a scale of 0 = “never” to 4 = “at least every day” and whether they were concerned about this (e.g., “I worried how being online affected my mental health”) rated on a scale of 0 = “not at all” to 4 = “very much”.<br><br>Parents will complete the parent version of **My Life Online** (MYLO). Parents will rate how much being online has affected their child’s everyday life in the past two weeks (e.g., “My child missed meals”, “My child had difficulties at school”). Responses will be provided on a scale ranging from “never” = 0 to “at least every day” = 4. They will also rate perceived positive and negative impacts of their child’s online activity on their mental health and	A, P	A, P	A, P

		relationships (e.g., "Being online positively affected my child's mental health" or "My child's online activity damaged our relationship"). Responses will be provided on a 5-point scale ranging from "not at all" = 0 to "very much" = 4. After reverse coding responses to items regarding positive impacts, item scores will be summed to derive an overall negative impact score.			
	Self-management of digital activity	In section two of MYLO adolescents (14 items) will rate the extent they tried to reduce the negative effects of their digital activity (e.g., "Took a break from social media", "Removed or blocked accounts"). Responses are provided on a scale ranging from 0 = "never" to 4 = "at least every day".	A	A	A
Individual characteristics	Social comparison	**Social Comparison Scale** (SCS; Allan & Gilbert, 1995). Adolescents will rate how they compare themselves to others using 10 pairs of opposite characteristics (e.g., "inferior-superior", "weaker-stronger") on a scale ranging from 1 to 10. Individual item scores are summed to derive an overall score where a higher score represents a more favourable comparison of oneself in respect to others.	A	A	A
	Social desirability	The **Children's Social Desirability – Short Scale** (CSD-SS; Miller et al., 2014). Adolescents will answer "yes" or "no" to each of 14 items and a higher score represents a greater tendency to answer in a more socially desirable manner.	A		
	Self-efficacy	The 10-item **Generalised Self-Efficacy Scale** (GSES; Schwarzer & Jerusalem, 1995), which measures one's perceptions of their ability to perform novel or difficult tasks, or cope with adversity. Responses are provided on a 4-point scale ranging from 1 = "not at all true" to 4 = "exactly true" and are summed to derive an overall score.	A	A	A
	Resilience	The **Youth Online and Offline Resilience Scale** (YOORS; adapted from Smith et al., 2008). This 8-item measure includes the original 6 items of the Brief Resilience Scale measuring domain-general resilience and two new items relating specifically to resilience online (e.g., "When something upsets me online, I tend to get over it quickly"). Responses are made on a 5-point scale ranging from 1 = "strongly disagree" to 5 = "strongly agree".	A	A	A
	Emotion regulation	**Emotion Regulation Questionnaire for Children and Adolescents** (ERQ-CA; Gullone & Taffe, 2012). The ERQ-CA measures two regulation strategies with the respective subscale scores. Cognitive reappraisal subscale (6 items) measures the extent to which an individual actively attempts to change how they think about a situation to change its emotional impact. Expressive suppression subscale (4 items) measures the extent to which an individual inhibits the behavioural expression of their emotions (Gross & John, 2012). Responses are made on a 7-point scale, ranging from 1 = "strongly disagree" to 7 = "strongly agree" and summed for the respective subscales. Higher scores indicated higher usage of the strategy.	A	A	A

Contextual factors	Social support	Social support will be measured with the **Social Support Questionnaire - 6** (SCQ; Sarason et al., 1987). This 12-item questionnaire measures availability and satisfaction with support. To measure the availability of social support, adolescents will state how many people (ranging from “0” to “7 and more”) they can count on to receive various forms of support. Then they rate how satisfied they are with the support they receive using a 6-point rating scale ranging from “very dissatisfied” to “very satisfied”.	A	A	A
	Loneliness	Loneliness will be measured with the 4-item **UCLA Loneliness Questionnaire** (Russell et al., 1980). Responses are made on 4-point scale ranging from 0 = ‘never’ to 3 = “often’ and individual item scores are summed to derive an overall loneliness score.	A	A	A
	Offline experiences and events	Frequency of offline experiences will be measured with the newly developed **Personal Experiences in Everyday Life** (PEEL) questionnaire. This is a new 19-item measure consisting of positive (e.g., hobbies, exercise) and negative (e.g., drinking alcohol, missing school) offline experiences and activities. Responses are provided on a 5-point scale ranging from 0 = “never” to 4 = “at least every day”.	A	A	A
	Substantial life events	The presence of significant life events in adolescence (e.g., moving home, parental job loss, new sibling) will be measured with a 10-item questionnaire adapted from the **Adverse Life Events Checklist** (Tiet et al., 2001). Adolescents will respond “yes’ = 1 or “no” = 0 and responses will be summed to derive an overall score.	A	A	A
Parental mental health and wellbeing	Depression symptoms	**The Patient Health Questionnaire** (PHQ-9; Kroenke et al., 2001). The PHQ-9 assesses depression symptoms using a 4-point scale ranging from “not at all” = 0 to “nearly every day” = 3. Individual items scores are summed to derive an overall score - higher overall score represents more severe depression symptoms.	P	P	P
	Wellbeing	Wellbeing will be measured with the WEMWBS.	P	P	P
Other parental measures	Child-parent relationship	The **Child-Parent Relationship Scale – Short Form** (CPRS-SF; Driscoll & Pianta, 2011), which includes two subscales: the 7-item closeness subscale scale and the 8-item conflict subscale. Parents will rate how much each statement applies to their relationship with their child on a 5-point rating scale ranging from “definitely does not apply” = 1 to “definitely applies” = 5.	P	P	P
