# Supplementary material for: Dynamic Interplay of Online Risk and Resilience in Adolescence (DIORA): a protocol for a 12-month prospective observational study testing the associations among digital activity, affective and cognitive reactions and depression symptoms in a community sample of UK adolescents
Source: BMJ Open. 2024 Sep 30;14(9):e085061. doi: 10.1136/bmjopen-2024-085061 (PMC11448176; doi:10.1136/bmjopen-2024-085061)
Supplement: Author's manuscript [file bmjopen-2024-085061.draft_revisions.pdf]

BMJ Open is committed to open peer review. As part of this commitment we make the peer review history of every article we publish publicly available.

When an article is published we post the peer reviewers' comments and the authors' responses online. We also post the versions of the paper that were used during peer review. These are the versions that the peer review comments apply to.

The versions of the paper that follow are the versions that were submitted during the peer review process. They are not the versions of record or the final published versions. They should not be cited or distributed as the published version of this manuscript.

BMJ Open is an open access journal and the full, final, typeset and author-corrected version of record of the manuscript is available on our site with no access controls, subscription charges or pay-per-view fees (http://bmjopen.bmj.com).

If you have any questions on BMJ Open's open peer review process please email info.bmjopen@bmj.com

BMJ Open

Dynamic Interplay of Online Risk and Resilience in Adolescence (DIORA): Protocol for a 12-month longitudinal study of the associations between digital activity, affective and cognitive reactions and depression during adolescence

Journal:	*BMJ Open*
Manuscript ID	bmjopen-2024-085061
Article Type:	Protocol
Date Submitted by the Author:	05-Feb-2024
Complete List of Authors:	Kostyrka-Allchorne, Katarzyna; Queen Mary University of London<br>Stoilova, Mariya; London School of Economics and Political Science, Department of Media and Communications<br>Bourgaize, Jake; King's College London, Department of Child & Adolescent Psychiatry<br>Murray, Aja; The University of Edinburgh, Department of Psychology<br>Azeri, Eliz; King's College London, Department of Psychology<br>Hollis, Chris; University of Nottingham, Division of Psychiatry<br>Townsend, Ellen; University of Nottingham, School of Psychology<br>Livingstone, Sonia; The London School of Economics and Political Science<br>Sonuga-Barke, Edmund; King's College London, Department of Child & Adolescent Psychiatry; Aarhus University, Department of Child & Adolescent Psychiatry<br>Youth, Digital; University of Nottingham
Keywords:	Adolescent, Depression & mood disorders < PSYCHIATRY, Behavior

SCHOLARONE™ Manuscripts

Dynamic Interplay of Online Risk and Resilience in Adolescence (DIORA): Protocol for a 12-month longitudinal study of the associations between digital activity, affective and cognitive reactions and depression during adolescence

Katarzyna Kostyrka-Allchorne[1,2], Mariya Stoilova[3], Jake Bourgaize[2], Aja Murray[4], Eliz Azeri[5], Chris Hollis[6], Ellen Townsend[6], Sonia Livingstone[3]* & Edmund J.S. Sonuga-Barke[2,7]* on behalf of the Digital Youth research team

*joint senior authors.

1. Department of Psychology, School of Biological and Behavioural Sciences, Queen Mary University of London.
2. Department of Child & Adolescent Psychiatry, Institute of Psychiatry, Psychology & Neuroscience, King's College London.
3. Department of Media and Communications, London School of Economics and Political Science.
4. Department of Psychology, University of Edinburgh
5. Department of Psychology, Institute of Psychiatry, Psychology & Neuroscience, King's College London.
6. Institute of Mental Health, University of Nottingham.
7. Department of Child & Adolescent Psychiatry, Aarhus University, Denmark.

Correspondence: Professor Edmund Sonuga-Barke edmund.sonuga-barke@kcl.ac.uk

For peer review only - http://bmjopen.bmj.com/site/about/guidelines.xhtml

1 Abstract

Introduction

The impact of digital activity on adolescent mental health has been difficult to assess because of methodological limitations and a lack of strong theory. *Dynamic Interplay of Online Risk and Resilience in Adolescence* (DIORA) is a longitudinal study designed to address these core limitations and tease apart the reciprocal influences linking digital activity and depression symptoms (hereafter 'depression') over 12 months in middle adolescence. This study will examine whether negative affective and cognitive reactions evoked by risky digital activity increase depression. It will also examine whether protective characteristics (e.g., resilience) moderate the associations between digital activity and depression. DIORA will also explore the reverse pathway from digital activity to depression - specifically, whether depression can either further exacerbate negative affective and cognitive reactions and, in turn, increase risky digital activity or risks can be mitigated through active management of digital activity and/or reactions that it evokes. Finally, the study will examine whether the effects of digital activity observed for depression contrast with those observed for wellbeing.

Methods and analysis

This is a prospective observational study with three assessment points: baseline (T1), 6 months (T2), and 12 months (T3). We aim to recruit a minimum of 276 adolescents aged between 13 and 14 years from secondary schools in the United Kingdom and one parent/caregiver/guardian (hereafter, 'parent') for each adolescent. Study questionnaires will be completed online.

We will fit a range of models to examine the direct and indirect associations between digital activity, the reactions it evokes, depression and wellbeing, and individual and contextual mediators and moderators drawing on the structural equation modelling framework.

For peer review only

Ethics and dissemination

The study was approved by the London School of Economics and Political Science Research Ethics Committee, reference number 249287. Results will be published in peer-reviewed scientific journals and disseminated through presentations, posters and blogs.

2 Strengths and limitations

5 bullet points relating specifically to the methods - not the results of the study.

- We move beyond 'screen time' or 'amount of social media use' by characterising digital activity to distinguish its distinctive risk and protective characteristics. We also recognise that the effects of digital activity may depend on the affective and cognitive reactions it evokes. Therefore, we use the ***Digital Activities and Feelings Inventory*** (DAFI), which separately measures digital activities and the reactions that these activities evoke.

- We selected depression as our primary outcome because adolescence is a period of increased risk of depression and the theoretical understanding of the relationship between life events and experiences and depression is well developed. It is also important to examine the effects on positive mental states, so psychological wellbeing is included as a secondary outcome.

- Given the possibility that the meaning and impact of digital activity are likely to vary considerably across adolescence, we chose to limit our focus to mid-adolescence, a period when the risk of depression is beginning to increase. This provides the statistical power required to explore the relationship between digital activity and mental health within our chosen developmental period.

For peer review only - http://bmjopen.bmj.com/site/about/guidelines.xhtml

- The study will adopt a longitudinal design to test the reciprocal relationship between digital activity and mental health, using cross-lagged models to explore nonlinear developmental trends. The time intervals were calibrated according to available research on the emergence of depression in adolescence, making it possible to examine the persistence of digital activity and evoked reactions across multiple time points, and model mediation using predictors, mediators and outcomes separated in time.

- It proved difficult to include measures that provide independent confirmation of adolescent self-reported digital activity or mental health. A strategy to obtain proxy measures from parents will be included but since parents will not receive any incentives for participation the results may be underpowered.

For peer review only

3 Introduction

Adolescence is a unique period of substantial biological, psychological and social changes. It is also a period that is associated with an increased risk of developing mental health problems, especially depression (1, 2). In Western countries, adolescent mental health problems have been steadily increasing over the past three decades. In the UK, for adolescents aged 17 to 19, rates of probable mental disorders rose from *1 in 10* in 2017 to *1 in 4* in 2022 (3). In parallel, there has been a consistent increase in the ownership of digital devices (e.g., smartphones, laptops, game consoles). For example, in the UK, 98% of 12-15-year-old adolescents own a mobile phone (4). Inevitably, digital activity has become an integral part of adolescent life with many adolescents reporting to be almost constantly online (5, 6). This has led to concerns that engaging in digital activity may be harmful to adolescent mental health and psychological wellbeing (7).

For peer review only - http://bmjopen.bmj.com/site/about/guidelines.xhtml

However, research findings are nuanced (8), with small effect sizes (9), and subject to measurement problems (10) that require careful contextualisation (11). Systematic reviews of the literature on the link between digital activity and mental health reveal mixed and inconsistent results (7, 12-15). Specifically, results vary depending on the type of content and users' mood and susceptibility (16), as well as on their vulnerability and available support (17). At the same time, the potential benefits of digital activity have been highlighted, including opportunities for social connection, support and entertainment (18, 19) and greater control over how one selects and engages with the external environment (20). Indeed, research identifies the benefits of digital activity, though few studies attempt or find links with robust measures of positive mental health or wellbeing (21, 22). Literature reviews often call for more specificity in the construction of measures and research designs.

Finding adequate measures of digital activity has proved particularly challenging, with many studies using a simple measure of 'screen time' or 'social media use' (23). This is important because different digital activities might be linked to different mental health outcomes (24). Mental health outcome measures can also be problematic, with some studies failing to distinguish between different mental health conditions, even though the effects may differ for anxiety and depression (25) and the effects for wellbeing may not simply be the inverse of those for depression (26). Regarding research design, prior studies have often mixed participants of different ages or developmental stages, although the effects may be developmentally specific (27). However, the most criticised aspect of prior designs is reliance on cross-sectional data even though it is vital to clarify the causal direction: does digital activity increase mental health risks or does mental health determine digital activity (28)? Related to this issue of causality, is locating the effects of digital activity in the context of offline risk and protective factors. For example, controlling for confounding risks may change the digital activity-

For peer review only - http://bmjopen.bmj.com/site/about/guidelines.xhtml

mental health relationship (29-31). Other potential mediating or moderating mechanisms may also drive or alter the relationship between digital activity and mental health (32).

To address these limitations, the present study, which is funded by the Medical Research Council as part of a large multi-institutional programme of research called Digital Youth https://digitalyouth.ac.uk (funder reference MR/W002450/1; Chief Investigators: Professors Hollis and Townsend, University of Nottingham), aims to examine the reciprocal longitudinal direct and indirect pathways between adolescent mental health and wellbeing and digital activity.

The study was designed to test a recently proposed cognitive-affective theory of the complex and reciprocal relationships between digital activity and depression (33). In this study, we propose three core hypotheses.

First, digital activity impacts depression through the frequency and persistence of the negative reactions it evokes in individuals. These encompass both negative affect (e.g., anger, annoyance) and negative cognitions (e.g., "I am stupid", "I am inferior").

Second, depression will, in turn, exacerbate digital risk exposures and experiences (i.e., leading individuals to engage in more negative digital activity) or the amount of negative affective and cognitive reactions it evokes, creating the potential for destructive cycles that may alter the course of development.

Third, individuals who recognise the negative impact of digital activity on their mental health might be able to mitigate it by either reducing their risky digital activity and/or by modifying their reactions to it. This, in turn, will lead to better mental health outcomes.

For peer review only - http://bmjopen.bmj.com/site/about/guidelines.xhtml

The *primary objective* of this study is to examine whether digital activity predicts an increase in depression symptoms over a 12-month period in middle adolescence (i.e., age 13-14 years), the developmental period when depression risk is beginning to increase (34).

Secondary objectives are to examine:

1. Whether depression is prospectively associated with risky digital activity over a 12-month period.
2. Whether the pathway from digital activity to depression is mediated by affective and cognitive reactions evoked by digital activity.
3. Whether, reciprocally, depression feeds back to alter patterns of digital activity and the reactions it evokes.
4. Whether personal resilience and other known risk and protective factors moderate the pathway from digital activity to depression and vice versa.
5. Whether effects observed for wellbeing are the inverse of those observed for depression.
6. Explore whether an individual's concerns about the impact their digital activity is having on their mental health leads to modifying their pattern of digital activity and/or their reactions to it.

We will also explore which factors predict high-risk digital activity and what proportion of adolescents are concerned about the impact of digital activity on their lives and mental health. What proportion attempts to modify their activity in light of this? What approaches do they use?

For peer review only - http://bmjopen.bmj.com/site/about/guidelines.xhtml
For peer review only

4 Methods and analysis

4.1 Study design

This is a longitudinal observational study with measures taken at baseline (T1), 6 months (T3), and 12 months (T3) post-baseline.

4.2 Study setting

All study procedures (consent form and questionnaire completion) will be completed remotely using Qualtrics. Participants will either complete the study measures at school or use their own devices connected to the internet in their preferred setting.

4.3 Participant selection and eligibility criteria

Participants in the study will be adolescents and their parents. Adolescents will be recruited from secondary schools in the United Kingdom. Each adolescent will nominate a parent (caregiver/guardian), who will also be invited to participate. To ensure participants represent a wide socioeconomic spectrum, we will approach a range of schools including mainstream and alternative provision schools, either state-funded or independent. To identify schools, we will use contacts in our professional networks as well as information from the Department for Education https://get-information-schools.service.gov.uk. We will continue approaching schools with a request to support recruitment until we have sufficient adolescents consented to the study. A member of the research team will meet with each school's nominated representative to discuss the study.

4.3.1 Inclusion criteria

- Adolescents attending a secondary school in England, Wales, or Scotland.
- The adolescents are aged 13-14 years old at the time of enrolment (typically, Year 9 in England and Wales or S3 in Scotland).

For peer review only - http://bmjopen.bmj.com/site/about/guidelines.xhtml

- Adolescents with access to a digital device connected to the internet (e.g., owned or shared at home, or a publicly shared device).
- There are no specific inclusion criteria for parents.

4.3.2 Exclusion criteria

- Adolescents will be excluded if they are unable to complete assessments independently.

4.4 Sample size justification

We judged the minimum clinically meaningful effect size of interest to detect, based on potential public health significance, to be r = .2. Our cross-sectional pilot study (publication forthcoming) found correlations with much larger effect sizes than this between proposed predictors and outcomes – suggesting that this effect size is obtainable. An *a priori* biserial normal model was used to calculate the required sample size (35). To achieve 80% power for an alpha of .05, this power analysis indicated a sample size of 193 is needed. Assuming the conservative 70% retention at 12-month follow-up, we will recruit a minimum of n = 276 adolescents; however, we will aim for the sample to be larger to allow sufficient power for moderation/mediation analyses.

4.5 Recruitment and follow-up procedures

Researchers will work with schools to raise adolescents' and parents' awareness of the study and to provide opportunities for asking questions. This may involve visiting schools to talk about the study during assemblies/classes, asking schools to display posters and share flyers with eligible students, and circulating study information via the school's newsletter, website, and social media.

Schools will also be asked to share a digital recruitment pack with each adolescent, which will include study promotion materials (e.g., a flyer) and information sheets for adolescents and parents.

For peer review only - http://bmjopen.bmj.com/site/about/guidelines.xhtml

The information sheet will include a link (e.g., a QR code) to an online consent form for the adolescent.

The school will also choose one of the two alternative models regarding parental consent, that is, the opt-in and opt-out models. Under the opt-in model, adolescents will not be able to participate unless the parent (or legal guardian) actively consents to their child's participation by filling in an online consent form. Under the opt-out model, adolescents will be asked to seek verbal consent from their parent (or legal guardian) and will be able to participate unless their parent (or legal guardian explicitly chooses for their child not to participate in the study. Under both consent models, adolescents and parents will need to consent to their own participation.

Adolescents and their parents will provide consent online. Following consent, adolescents and parents will receive a notification via email and/or text message to complete the battery of T1 questionnaires online. Each assessment window will close after three weeks of opening. A notification to complete assessments will be sent via email and/or text message on the day that the assessment is due to be completed.

Each adolescent will receive a £15 voucher for completing the questionnaires at each time point and a 'bonus' of £15 for completing assessments at all timepoints (a total of £60 for those who complete the whole study). There will be no compensation for parents. Each school facilitating recruitment will receive between £100 and £250 to thank them for their support (amount depending on the level of support given to the study's recruitment). Other non-monetary incentives (e.g., psychology talks, school-level feedback, etc.) will be negotiated with individual schools.

4.6 Participant timeline

Adolescent and parent measures will be completed at T1, T2, and T3. A detailed schedule of assessments is presented in Table 1.

For peer review only - http://bmjopen.bmj.com/site/about/guidelines.xhtml

4.7 Outcomes

4.7.1 Adolescent-reported outcomes

Adolescents will report their depression and anxiety symptoms using the Revised Child Anxiety and Depression Scale 25–Youth Version (RCADS-25; 36). Conduct problems and attention-deficit hyperactivity symptoms will be measured with the respective subscales of The Strengths and Difficulties Questionnaire (SDQ; 37). Wellbeing will be measured with the Warwick-Edinburgh Mental Wellbeing Scale (WEMWBS; 38).

Adolescents will also answer a single question about screen time and complete a detailed assessment of their digital activity using the recently developed *My Online Activity* subscale of the *Digital Activity and Feelings Inventory* (DAFI). Reactions evoked by digital activity will be measured with the *My Feelings Online* subscale of the DAFI. The impact of online activities on daily life and management behaviours will be measured by a second new measure: the *My Life Online* (MYLO) questionnaire. Finally, we will measure the following individual characteristics: psychological resilience, social comparison, self-efficacy, emotion regulation and social desirability and the following contextual factors: offline events and experiences, loneliness, social support and the presence of significant life events. A demographic questionnaire will ask for information about adolescents' age, biological sex and gender identity, ethnicity, eligibility for free school meals, and perceived family affluence. A detailed description of the measures is provided in Table 1.

4.7.2 Parent-reported outcomes

Parents will report their child's depression and anxiety symptoms on the respective subscales of the RCADS-25. Adolescent psychological wellbeing will be measured with an adapted version of the WEMWBS. Parents will also complete a parent-report version of MYLO to rate how much being online has affected their child's everyday life and will rate perceived positive and negative impacts of

For peer review only - http://bmjopen.bmj.com/site/about/guidelines.xhtml

their child's online activity on their mental health and relationships. They will also report on their relationship with their child on the Child-Parent Relationship Scale – Short Form (39).

Parental depression will be measured with The Patient Health Questionnaire (40) and wellbeing will be measured with the WEMWBS.

To obtain demographic information, parents will provide information about their own ethnicity, education and employment status, as well as report the occupation of the main earner in the family. The latter will be categorised using the nine major groups from the Office for National Statistics Standard Occupational Classification Hierarchy (41).

[Here insert Table 1 "Table 1: An overview of the study outcomes and their measures completed by adolescents and their parents at each timepoint"]

4.8 General procedure

4.8.1 Data collection methods

Outcome data will be collected online using Qualtrics - a secure dedicated survey platform. Each consenting participant (i.e., adolescent and parent) will receive a link to the relevant T1 questionnaires. If a participant does not start the T1 questionnaires within three weeks, it will be assumed they do not wish to continue and will not be invited to complete follow-up assessments.

Participants who completed T1 questionnaires will be sent two follow-up assessment invitations (T2 and T3) via text message or email. At each timepoint, they will have three weeks to start completing the questionnaires and seven days to finish the questionnaires once started. Up to three reminders will be sent to those who have not completed the assessment.

For peer review only - http://bmjopen.bmj.com/site/about/guidelines.xhtml

4.8.2 Withdrawals

Participants will be free to withdraw at any point in the study by either ignoring invitations to complete the online questionnaires or by emailing the research team. Data collected before withdrawal will be included in the analysis.

4.9 Public patient involvement and engagement: youth panel

The overall Digital Youth programme research priorities, ethical and design considerations were considered at the pre-funding stage through the consultation with the MRC Adolescence, Mental Health & Developing Mind Engagement Award Young Person Advisory Group (now Sprouting Minds) - a youth panel which informs and supports Digital Youth researchers through co-facilitation and collaboration.

In addition, DIORA has a dedicated youth advisory panel comprising adolescents aged 13-17, which meets online at least every two months. The DIORA youth panel played a crucial role in the development of the DAFI to ensure that a list of digital activities and reactions they induce were relevant to adolescents. The panel also reviewed and advised on current study plans and procedures and participant-facing documents to ensure these are acceptable and inclusive. For example, the panel helped to short-list digital activities that are relevant to contemporary adolescents and advised that recruitment occurs through schools to increase the perceived legitimacy of research (also because students may not trust or know the academic institutions that are conducting the study). We will continue working with both Sprouting Minds and the DIORA youth panel throughout this project and future activities will include reviewing the progress of the longitudinal study, interpreting findings, and involvement in results dissemination.

For peer review only - http://bmjopen.bmj.com/site/about/guidelines.xhtml

4.10 Analysis plan

We will examine information about the proportion of missing data and its patterns. Summary statistics will be used to describe demographic information and provide information about the level of psychopathology symptoms and digital activity in the adolescent sample.

We will fit a range of models to examine the associations between digital activity, depression and wellbeing drawing on the structural equation modelling framework, which provides the flexibility to implement a diversity of models which map to the study's objectives. For example, (random-intercept) cross-lagged panel models will be used to examine (potentially reciprocal) directional relations over time between digital activity and depression; growth curve models will be used to examine trajectories over time, their individual differences, and predictors of these individual differences; and mediating paths can be added to models to examine indirect effects or 'mechanistic pathways'. Further details of the analyses will be specified in the statistical analysis plan.

5 Ethics and dissemination

5.1 Ethics approval and safety consideration

The study was approved by the London School of Economics Research Ethics Committee, reference number 249287. Any future protocol amendments will be documented and submitted for ethical approval prior to implementation.

Given the observational design, the risk of harm to participants is minimal. There is a small likelihood that completing study questionnaires may result in new concerns related to parent or child mental health, digital activity, relationships, or affective and cognitive reactions. All questionnaires/procedures have been reviewed by the DIORA youth panel to ensure sensitivity and acceptability to adolescents and their parents. Participants will be able to skip questions they do not

For peer review only

want to answer, and all participants will be provided with information on seeking mental health support and contacts for national crisis services at the start and end of each assessment battery.

5.2 Dissemination

Results will be published in a peer-reviewed scientific journal and included in the funder report. Authorship will be determined according to the Digital Youth's publication policy developed on the basis of the Committee on Publication Ethics (COPE) standards based on individual contributions. Findings will be disseminated through presentations at conferences and seminars in the UK and overseas. There will also be a general dissemination through the Digital Youth and institutional websites, social media, and stakeholder engagement activities.

5.3 Study oversight

Overall programme operational oversight and progress monitoring is provided by the Digital Youth leadership team. This group comprises the programme Principal Investigators (Profs. Hollis and Townsend), work package leads, Programme Manager and members of Sprouting Minds. Overall scientific oversight is provided by the Scientific Advisory Board which reviews and approves study protocols and receives progress updates. This comprises international academic experts representing disciplines relevant to the programme (e.g., adolescent mental health, computer science, digital psychological interventions).

6 Study status

The study recruitment started in October 2023 with data collection planned to be completed in March 2025.

For peer review only - http://bmjopen.bmj.com/site/about/guidelines.xhtml

7 Funding

The authors (MS, KKA, JB, EA, CH, ET, SL and ESB) acknowledge the support of the UK Research and Innovation (UKRI) Digital Youth Programme award [MRC project reference MR/W002450/1] which is part of the AHRC/ESRC/MRC Adolescence, Mental Health and the Developing Mind programme. The project funding covers all costs in relation to the study, including but not limited to, higher education institutions' costs, equipment, and the youth panel costs. The funding covers the period from 1 September 2021 to 31 August 2025.

8 Author contributions

CH and ET are the overall Digital Youth programme chief investigators. SL, ESB, MS, JB, AM and KKA contributed to the design of the study. AJ, ESB and SL contributed to the analysis plans. MS and JB prepared the protocol and other study documentation for ethical approval. KKA and MS prepared the first draft of the manuscript for publication. EA, JB and NN organised the activities of the DIORA youth panel and liaised with Sprouting Minds. All authors contributed to drafting and revising the manuscript and approved the final version to be submitted for publication.

9

References

1. Weavers B, Riglin L, Martin J, Anney R, Collishaw S, Heron J, et al. Characterising depression trajectories in young people at high familial risk of depression. Journal of Affective Disorders. 2023;337:66-74.
2. Wiederhold BK. The escalating crisis in adolescent mental health. Mary Ann Liebert, Inc., publishers 140 Huguenot Street, 3rd Floor New …; 2022. p. 81-2.
3. Newlove-Delgado T, Marcheselli F, Williams T, Mandalia D, Davis J, McManus S, et al. Mental Health of Children and Young People in England, 2022-wave 3 follow up to the 2017 survey. 2022.
4. Ofcom. Children and parents: media use and attitudes report 2023. 2023 29 March 2023.
5. Nagata JM, Cortez CA, Cattle CJ, Ganson KT, Iyer P, Bibbins-Domingo K, et al. Screen time use among US adolescents during the COVID-19 pandemic: findings from the adolescent brain cognitive development (ABCD) study. JAMA pediatrics. 2022;176(1):94-6.
6. Thomas G, Bennie JA, De Cocker K, Castro O, Biddle SJ. A descriptive epidemiology of screen-based devices by children and adolescents: a scoping review of 130 surveillance studies since 2000. Child Indicators Research. 2020;13:935-50.
7. Odgers CL, Jensen MR. Annual research review: Adolescent mental health in the digital age: Facts, fears, and future directions. Journal of Child Psychology and Psychiatry. 2020;61(3):336-48.

For peer review only - http://bmjopen.bmj.com/site/about/guidelines.xhtml

8. Ferguson CJ. Does the internet make the world worse? Depression, aggression and polarization in the social media age. Bulletin of Science, Technology & Society. 2021;41(4):116-35.
9. Steinsbekk S, Nesi J, Wichstrøm L. Social media behaviors and symptoms of anxiety and depression. A four-wave cohort study from age 10–16 years. Computers in Human Behavior. 2023;147:107859.
10. Parry DA, Davidson BI, Sewall CJ, Fisher JT, Mieczkowski H, Quintana DS. A systematic review and meta-analysis of discrepancies between logged and self-reported digital media use. Nature Human Behaviour. 2021;5(11):1535-47.
11. Panayiotou M, Black L, Carmichael-Murphy P, Qualter P, Humphrey N. Time spent on social media among the least influential factors in adolescent mental health: preliminary results from a panel network analysis. Nature Mental Health. 2023;1(5):316-26.
12. Orben A. Teenagers, screens and social media: a narrative review of reviews and key studies. Social Psychiatry and Psychiatric Epidemiology. 2020;55(4):407-14.
13. Keles B, McCrae N, Grealish A. A systematic review: the influence of social media on depression, anxiety and psychological distress in adolescents. International journal of adolescence and youth. 2020;25(1):79-93.
14. Kostyrka-Allchorne K, Stoilova M, Bourgaize J, Rahali M, Livingstone S, Sonuga-Barke E. Digital experiences and their impact on the lives of adolescents with pre-existing anxiety, depression, eating and nonsuicidal self-injury conditions–a systematic review. Child and Adolescent Mental Health. 2023;28(1):22-32.
15. Sanders T, Noetel M, Parker P, Del Pozo Cruz B, Biddle S, Ronto R, et al. An umbrella review of the benefits and risks associated with youths' interactions with electronic screens. Nature Human Behaviour. 2023.
16. Valkenburg PM, van Driel II, Beyens I. The associations of active and passive social media use with well-being: A critical scoping review. New media & society. 2022;24(2):530-49.
17. El-Asam A, Lane R, Katz A, editors. Psychological distress and its mediating effect on experiences of online risk: The case for vulnerable young people. Frontiers in Education; 2022: Frontiers.
18. Hollis C, Livingstone S, Sonuga-Barke E. The role of digital technology in children and young people's mental health–a triple-edged sword? : Wiley Online Library; 2020. p. 837-41.
19. Anthony R, Young H, Hewitt G, Sloan L, Moore G, Murphy S, et al. Young people's online communication and its association with mental well-being: results from the 2019 student health and well-being survey. Child and Adolescent Mental Health. 2023;28(1):4-11.
20. Sonuga-Barke E, Kostyrka-Allchorne K. Editorial Perspective: Attention-deficit/hyperactivity disorder viewed as neuro-divergence in the digital world. Journal of Child Psychology and Psychiatry. 2023;64(6):972-4.
21. O'Reilly M. Social media and adolescent mental health: the good, the bad and the ugly. Journal of Mental Health. 2020;29(2):200-6.
22. Halbrook YJ, O'Donnell AT, Msetfi RM. When and how video games can be good: A review of the positive effects of video games on well-being. Perspectives on Psychological Science. 2019;14(6):1096-104.
23. Browne DT, May SS, Colucci L, Hurst-Della Pietra P, Christakis D, Asamoah T, et al. From screen time to the digital level of analysis: a scoping review of measures for digital media use in children and adolescents. BMJ open. 2021;11(5):e046367.
24. Tang S, Werner-Seidler A, Torok M, Mackinnon AJ, Christensen H. The relationship between screen time and mental health in young people: A systematic review of longitudinal studies. Clinical psychology review. 2021;86:102021.
25. Kim S, Favotto L, Halladay J, Wang L, Boyle MH, Georgiades K. Differential associations between passive and active forms of screen time and adolescent mood and anxiety disorders. Social psychiatry and psychiatric epidemiology. 2020;55:1469-78.
26. Stiglic N, Viner RM. Effects of screentime on the health and well-being of children and adolescents: a systematic review of reviews. BMJ open. 2019;9(1):e023191.
27. El Asam A, Samara M, Terry P. Problematic internet use and mental health among British children and adolescents. Addictive behaviors. 2019;90:428-36.
28. Boer M, Stevens GW, Finkenauer C, de Looze ME, van den Eijnden RJ. Social media use intensity, social media use problems, and mental health among adolescents: Investigating directionality and mediating processes. Computers in Human Behavior. 2021;116:106645.
29. Jensen M, George MJ, Russell MR, Odgers CL. Young adolescents' digital technology use and mental health symptoms: Little evidence of longitudinal or daily linkages. Clinical Psychological Science. 2019;7(6):1416-33.

For peer review only - http://bmjopen.bmj.com/site/about/guidelines.xhtml

30. Schemer C, Masur PK, Geiß S, Müller P, Schäfer S. The impact of internet and social media use on well-being: A longitudinal analysis of adolescents across nine years. Journal of Computer-Mediated Communication. 2021;26(1):1-21.
31. Paulich KN, Ross JM, Lessem JM, Hewitt JK. Screen time and early adolescent mental health, academic, and social outcomes in 9- and 10- year old children: Utilizing the Adolescent Brain Cognitive Development ℠ (ABCD) Study. PLoS One. 2021;16(9):e0256591.
32. Granic I, Morita H, Scholten H. Beyond screen time: Identity development in the digital age. Psychological Inquiry. 2020;31(3):195-223.
33. Sonuga-Barke E, Stoilova M, Kostyrka-Allchorne K, Bourgaize J, Hollis C, Townsend E, et al. Adolescents' digital activity and depression: Outlining a developmental framework integrating risk, reactivity, resilience and reciprocity. 2023.
34. Grossberg A, Rice T. Depression and suicidal behavior in adolescents. Medical Clinics. 2023;107(1):169-82.
35. Faul F, Erdfelder E, Buchner A, Lang A-G. Statistical power analyses using G* Power 3.1: Tests for correlation and regression analyses. Behavior research methods. 2009;41(4):1149-60.
36. Ebesutani C, Korathu-Larson P, Nakamura BJ, Higa-McMillan C, Chorpita B. The revised child anxiety and depression scale 25–parent version: scale development and validation in a school-based and clinical sample. Assessment. 2017;24(6):712-28.
37. Goodman R. The Strengths and Difficulties Questionnaire: a research note. Journal of child psychology and psychiatry. 1997;38(5):581-6.
38. Tennant R, Hiller L, Fishwick R, Platt S, Joseph S, Weich S, et al. The Warwick-Edinburgh Mental Well-being Scale (WEMWBS): development and UK validation. Health and Quality of Life Outcomes. 2007;5(1):63.
39. Driscoll K, Pianta RC. Mothers' and Fathers' Perceptions of Conflict and Closeness in Parent-Child Relationships during Early Childhood. Journal of Early Childhood & Infant Psychology. 2011(7).
40. Kroenke K, Spitzer RL, Williams JB. The PHQ-9: validity of a brief depression severity measure. J Gen Intern Med. 2001;16(9):606-13.
41. ONS. 2023 [Available from: https://onsdigital.github.io/dp-classification-tools/standard-occupational-classification/ONS_SOC_hierarchy_view.html.

For peer review only

For peer review only - http://bmjopen.bmj.com/site/about/guidelines.xhtml

Table 1. DIORA constructs, outcome, measures and schedule of assessments.

General construct	Outcome	Measure	T1	T2	T3
Demographic information		Adolescent age, biological sex, gender identity, ethnicity	A, P		
		Family affluence, eligibility for free school meals	A		
		Parental ethnicity, parental education, and employment status, main earner occupation.	P		
Adolescent psychopathology	Depression symptoms (primary outcome at T3)	**Revised Child Anxiety and Depression Scale 25–Youth Version** (RCADS-25; Ebesutani et al., 2017) subscale. Adolescents will rate how often each statement applies to them on a four-point scale (0 = never, 3 = always). The subscale score is derived by summing individual item scores. Higher scores on the subscale indicate greater symptoms of depression and/or anxiety.	A, P	A, P	A, P
	Anxiety symptoms	Anxiety subscale of the RCADS-25. For information about response scale and scoring see above.	A, P	A, P	A, P
	ADHD symptoms and conduct problems	Respective subscales of **The Strengths and Difficulties Questionnaire** (SDQ; Goodman, 1997). In this 25-item questionnaire adolescents will be provided with descriptions of personal characteristics and asked to indicate whether the description is true of themselves on a 3-point rating scale ranging from 0 = "not true" to 2 = "certainly true". After reverse coding, individual item scores are summed to derive an overall subscale score where the higher score represents greater severity of symptoms.	A		A
Adolescent wellbeing	Wellbeing	The 14-item **Warwick-Edinburgh Mental Wellbeing Scale** (WEMWBS; Tennant et al., 2007), which assesses the presence of positive thoughts and feelings, which relate to one's general functioning. All statements are positively worded and are rated on a 5-point rating scale ranging from 1 = "none of the time" to 5 = "all of the time". Individual item scores summed to calculate the total score.	A, P	A, P	A, P
Digital activity and screen time	Amount of time spent online	Screen time measured with a single question: "On an ordinary school day, about how long do you send on your phone or the internet (not counting time for schoolwork)?" Responses provided on a 9-point scale ranging from "little to no time" to "about 7 hours".	A	A	A

For peer review only - http://bmjopen.bmj.com/site/about/guidelines.xhtml

	Digital activity	*My Online Activity* subscale of the **Digital Activity and Feelings Inventory (DAFI);** measures four distinct dimensions of digital activity: high-risk content (7 items; e.g., “I watched some sad or dark content”), high-risk conduct (8 items; e.g., “I saw people talk about or show ways of being very thin”), entertainment (3 items; e.g. “I played games with others”), and social media use (4 items; e.g., “I like or shared other people’s posts”). Adolescents will rate how often they did or experienced each item in the past two weeks on a 5-point scale ranging from 0 = “never” to 4 = “at least every day”.	A	A	A
	Emotional reactions induced by digital activity	*My Feelings Online* subscale of the DAFI, which includes 10 positive and 10 negative feelings that encompass both general emotional reactions (e.g., “stressed”, “calm”) and those that specifically pertain to the self (e.g., “insecure”, “attractive”, “loved”). Adolescents will rate how often they experienced each item in relation to being online in the past two weeks on a 5-point scale ranging from 0 = “never” to 4 = “at least every day”. Information about the validation of this measure will be reported in the forthcoming publication.	A	A	A
	Impact of digital activity on daily functioning and concerns about the impact of digital activity on mental health and relationships	Adolescents will complete the **My Life Online** (MYLO) questionnaire. Section one (9 items), asks adolescents about how much their life was impacted by their digital activity (e.g., “I missed meals”, “I had difficulties at school/college/university”) rated on a scale of 0 = “never” to 4 = “at least every day” and whether they were concerned about this (e.g., “I worried how being online affected my mental health”) rated on a scale of 0 = “not at all” to 4 = “very much”.<br><br>Parents will complete the parent version of **My Life Online** (MYLO). Parents will rate how much being online has affected their child’s everyday life in the past two weeks (e.g., “My child missed meals”, “My child had difficulties at school”). Responses will be provided on a scale ranging from “never” = 0 to “at least every day” = 4. They will also rate perceived positive and negative impacts of their child’s online activity on their mental health and relationships (e.g., “Being online positively affected my child’s mental health” or “My child’s online activity damaged our relationship”). Responses will be provided on a 5-point scale ranging from "not at all” = 0 to “very much” = 4. After reverse coding responses to items regarding positive impacts, item scores will be summed to derive an overall negative impact score.	A, P	A, P	A, P
	Self-management of digital activity	In section two of MYLO adolescents (14 items) will rate the extent they tried to reduce the negative effects of their digital activity (e.g., “Took a break from social media”, “Removed or blocked accounts”). Responses are provided on a scale ranging from 0 = “never” to 4 = “at least every day”.	A	A	A

For peer review only - http://bmjopen.bmj.com/site/about/guidelines.xhtml

	Social comparison	**Social Comparison Scale** (SCS; Allan & Gilbert, 1995). Adolescents will rate how they compare themselves to others using 10 pairs of opposite characteristics (e.g., "inferior-superior", "weaker-stronger") on a scale ranging from 1 to 10. Individual item scores are summed to derive an overall score where a higher score represents a more favourable comparison of oneself in respect to others.	A	A	A
	Social desirability	The **Children's Social Desirability – Short Scale** (CSD-SS; Miller et al., 2014). Adolescents will answer "yes" or "no" to each of 14 items and a higher score represents a greater tendency to answer in a more socially desirable manner.	A		
	Self-efficacy	The 10-item **Generalised Self-Efficacy Scale** (GSES; Schwarzer & Jerusalem, 1995), which measures one's perceptions of their ability to perform novel or difficult tasks, or cope with adversity. Responses are provided on a 4-point scale ranging from 1 = "not at all true" to 4 = "exactly true" and are summed to derive an overall score.	A	A	A
	Resilience	The **Youth Online and Offline Resilience Scale** (YOORS; adapted from Smith et al., 2008). This 8-item measure includes the original 6 items of the Brief Resilience Scale measuring domain-general resilience and two new items relating specifically to resilience online (e.g., "When something upsets me online, I tend to get over it quickly"). Responses are made on a 5-point scale ranging from 1 = "strongly disagree" to 5 = "strongly agree".	A	A	A
	Emotion regulation	**Emotion Regulation Questionnaire for Children and Adolescents** (ERQ-CA; Gullone & Taffe, 2012). The ERQ-CA measures two regulation strategies with the respective subscale scores. Cognitive reappraisal subscale (6 items) measures the extent to which an individual actively attempts to change how they think about a situation to change its emotional impact. Expressive suppression subscale (4 items) measures the extent to which an individual inhibits the behavioural expression of their emotions (Gross & John, 2012). Responses are made on a 7-point scale, ranging from 1 = "strongly disagree" to 7 = "strongly agree" and summed for the respective subscales. Higher scores indicated higher usage of the strategy.	A	A	A
Contextual factors	Social support	Social support will be measured with the **Social Support Questionnaire - 6** (SCQ; Sarason et al., 1987). This 12-item questionnaire measures availability and satisfaction with support. To measure the availability of social support, adolescents will state how many people (ranging from "0" to "7 and more") they can count on to receive various forms of support. Then they rate how satisfied they are with the support they receive using a 6-point rating scale ranging from "very dissatisfied" to "very satisfied".	A	A	A
	Loneliness	Loneliness will be measured with the 4-item **UCLA Loneliness Questionnaire** (Russell et al., 1980). Responses are made on 4-point scale ranging from 0 = 'never' to 3 = "often' and individual item scores are summed to derive an overall loneliness score.	A	A	A

For peer review only

	Offline experiences and events	Frequency of offline experiences will be measured with the newly developed **Personal Experiences in Everyday Life** (PEEL) questionnaire. This is a new 19-item measure consisting of positive (e.g., hobbies, exercise) and negative (e.g., drinking alcohol, missing school) offline experiences and activities. Responses are provided on a 5-point scale ranging from 0 = "never" to 4 = "at least every day".	A	A	A
	Substantial life events	The presence of significant life events in adolescence (e.g., moving home, parental job loss, new sibling) will be measured with a 10-item questionnaire adapted from the **Adverse Life Events Checklist** (Tiet et al., 2001). Adolescents will respond "yes' = 1 or "no" = 0 and responses will be summed to derive an overall score.	A	A	A
Parental mental health and wellbeing	Depression symptoms	**The Patient Health Questionnaire** (PHQ-9; Kroenke et al., 2001). The PHQ-9 assesses depression symptoms using a 4-point scale ranging from "not at all" = 0 to "nearly every day" = 3. Individual items scores are summed to derive an overall score - higher overall score represents more severe depression symptoms.	P	P	P
	Wellbeing	Wellbeing will be measured with the WEMWBS.	P	P	P
Other parental measures	Child-parent relationship	The **Child-Parent Relationship Scale – Short Form** (CPRS-SF; Driscoll & Pianta, 2011), which includes two subscales: the 7-item closeness subscale scale and the 8-item conflict subscale. Parents will rate how much each statement applies to their relationship with their child on a 5-point rating scale ranging from "definitely does not apply" = 1 to "definitely applies" = 5.	P	P	P

For peer review only

STROBE Statement—Checklist of items that should be included in reports of ***cross-sectional studies***

	Item No	Recommendation	Page No
**Title and abstract**	1	(*a*) Indicate the study's design with a commonly used term in the title or the abstract	1, 2
		(*b*) Provide in the abstract an informative and balanced summary of what was done and what was found	2
**Introduction**			
Background/rationale	2	Explain the scientific background and rationale for the investigation being reported	4-6
Objectives	3	State specific objectives, including any prespecified hypotheses	7
**Methods**			
Study design	4	Present key elements of study design early in the paper	8
Setting	5	Describe the setting, locations, and relevant dates, including periods of recruitment, exposure, follow-up, and data collection	8
Participants	6	(*a*) Give the eligibility criteria, and the sources and methods of selection of participants	8-10
Variables	7	Clearly define all outcomes, exposures, predictors, potential confounders, and effect modifiers. Give diagnostic criteria, if applicable	10
Data sources/ measurement	8*	For each variable of interest, give sources of data and details of methods of assessment (measurement). Describe comparability of assessment methods if there is more than one group	*Table 1*
Bias	9	Describe any efforts to address potential sources of bias	N/A
Study size	10	Explain how the study size was arrived at	9
Quantitative variables	11	Explain how quantitative variables were handled in the analyses. If applicable, describe which groupings were chosen and why	N/A
Statistical methods	12	(*a*) Describe all statistical methods, including those used to control for confounding	14
		(*b*) Describe any methods used to examine subgroups and interactions	N/A
		(*c*) Explain how missing data were addressed	N/A
		(*d*) If applicable, describe analytical methods taking account of sampling strategy	N/A
		(*e*) Describe any sensitivity analyses	N/A
**Results**			
Participants	13*	(a) Report numbers of individuals at each stage of study—eg numbers potentially eligible, examined for eligibility, confirmed eligible, included in the study, completing follow-up, and analysed	N/A
		(b) Give reasons for non-participation at each stage	N/A
		(c) Consider use of a flow diagram	
Descriptive data	14*	(a) Give characteristics of study participants (eg demographic, clinical, social) and information on exposures and potential confounders	N/A
		(b) Indicate number of participants with missing data for each variable of interest	N/A
Outcome data	15*	Report numbers of outcome events or summary measures	N/A
Main results	16	(*a*) Give unadjusted estimates and, if applicable, confounder-adjusted estimates and their precision (eg, 95% confidence interval). Make clear which confounders were adjusted for and why they were included	N/A

For peer review only - http://bmjopen.bmj.com/site/about/guidelines.xhtml

		(*b*) Report category boundaries when continuous variables were categorized	N/A
		(*c*) If relevant, consider translating estimates of relative risk into absolute risk for a meaningful time period	N/A
Other analyses	17	Report other analyses done—eg analyses of subgroups and interactions, and sensitivity analyses	N/A
**Discussion**			
Key results	18	Summarise key results with reference to study objectives	N/A
Limitations	19	Discuss limitations of the study, taking into account sources of potential bias or imprecision. Discuss both direction and magnitude of any potential bias	N/A
Interpretation	20	Give a cautious overall interpretation of results considering objectives, limitations, multiplicity of analyses, results from similar studies, and other relevant evidence	N/A
Generalisability	21	Discuss the generalisability (external validity) of the study results	N/A
**Other information**			
Funding	22	Give the source of funding and the role of the funders for the present study and, if applicable, for the original study on which the present article is based	16

*Give information separately for exposed and unexposed groups.

Note: An Explanation and Elaboration article discusses each checklist item and gives methodological background and published examples of transparent reporting. The STROBE checklist is best used in conjunction with this article (freely available on the Web sites of PLoS Medicine at http://www.plosmedicine.org/, Annals of Internal Medicine at http://www.annals.org/, and Epidemiology at http://www.epidem.com/). Information on the STROBE Initiative is available at www.strobe-statement.org.

For peer review only

For peer review only - http://bmjopen.bmj.com/site/about/guidelines.xhtml

BMJ Open

Dynamic Interplay of Online Risk and Resilience in Adolescence (DIORA): a protocol for a 12-month prospective observational study testing the associations between digital activity, affective and cognitive reactions and depression symptoms in a community sample of UK adolescents

Journal:	*BMJ Open*
Manuscript ID	bmjopen-2024-085061.R1
Article Type:	Protocol
Date Submitted by the Author:	09-Sep-2024
Complete List of Authors:	Kostyrka-Allchorne, Katarzyna; Queen Mary University of London<br>Stoilova, Mariya; The London School of Economics and Political Science Department of Media and Communications,<br>Bourgaize, Jake; King’s College London, Department of Child & Adolescent Psychiatry<br>Murray, Aja; The University of Edinburgh, Department of Psychology<br>Azeri, Eliz; King’s College London, Department of Psychology<br>Hollis, Chris; University of Nottingham, Division of Psychiatry<br>Townsend, Ellen; University of Nottingham, School of Psychology<br>Livingstone, Sonia; The London School of Economics and Political Science<br>Sonuga-Barke, Edmund; King's College London<br>Youth, Digital; University of Nottingham
**Primary Subject Heading**:	Mental health
Secondary Subject Heading:	Paediatrics
Keywords:	Adolescent, Depression & mood disorders < PSYCHIATRY, Behavior, MENTAL HEALTH, Digital Technology

SCHOLARONE™ Manuscripts

Dynamic Interplay of Online Risk and Resilience in Adolescence (DIORA): a protocol for a 12-month prospective observational study testing the associations between digital activity, affective and cognitive reactions and depression symptoms in a community sample of UK adolescents

Katarzyna Kostyrka-Allchorne[1,2], Mariya Stoilova[3], Jake Bourgaize[2], Aja Murray[4], Eliz Azeri[5], Chris Hollis[6], Ellen Townsend[6], Sonia Livingstone[3]* & Edmund J.S. Sonuga-Barke[2,7]* on behalf of the Digital Youth research team

*joint senior authors.

1. Department of Psychology, School of Biological and Behavioural Sciences, Queen Mary University of London.
2. Department of Child & Adolescent Psychiatry, Institute of Psychiatry, Psychology & Neuroscience, King's College London.
3. Department of Media and Communications, London School of Economics and Political Science.
4. Department of Psychology, University of Edinburgh
5. Department of Psychology, Institute of Psychiatry, Psychology & Neuroscience, King's College London.
6. Institute of Mental Health, University of Nottingham.
7. Department of Child & Adolescent Psychiatry, Aarhus University, Denmark.

Correspondence: Professor Edmund Sonuga-Barke edmund.sonuga-barke@kcl.ac.uk

For peer review only - http://bmjopen.bmj.com/site/about/guidelines.xhtml

1 Abstract

Introduction

The impact of digital activity on adolescent mental health has been difficult to assess because of methodological limitations and a lack of strong theory. *Dynamic Interplay of Online Risk and Resilience in Adolescence* (DIORA) is a longitudinal study designed to address these core limitations and tease apart the reciprocal influences linking digital activity and depression symptoms (hereafter 'depression') over 12 months in middle adolescence. This study will examine whether negative affective and cognitive reactions evoked by risky digital activities increase depression. It will additionally examine whether protective characteristics (e.g., self-efficacy) moderate the associations between digital activity and depression. DIORA will also explore the reverse pathways between digital activity and depression, namely whether depression exacerbates negative affective and cognitive reactions and, in turn, increase risky digital activities or, further, whether risks can be mitigated through active management of digital activity and/or reactions that it evokes. Finally, the study will examine whether the effects of digital activity observed for depression contrast with those observed for wellbeing.

Methods and analysis

This is a prospective observational study with three assessment points: baseline (T1), 6 months (T2), and 12 months (T3). We aim to recruit a minimum of 276 adolescents aged between 13 and 14 years from secondary schools in the United Kingdom and one parent/caregiver/guardian (hereafter, 'parent') for each adolescent. Study questionnaires will be completed online.

We will fit a range of models to examine the direct and indirect associations between digital activity, the reactions it evokes, depression and wellbeing, and individual and contextual mediators and moderators drawing on the structural equation modelling framework.

For peer review only - http://bmjopen.bmj.com/site/about/guidelines.xhtml

Ethics and dissemination

The study was approved by the London School of Economics and Political Science Research Ethics Committee, reference number 249287. Results will be published in peer-reviewed scientific journals and disseminated through presentations, posters and blogs.

2 Strengths and limitations

5 bullet points relating specifically to the methods - not the results of the study.

- The DIORA study adopts a prospective longitudinal design to test the reciprocal relationships between digital activity and depression symptoms in a community sample of UK adolescents.
- We will improve on simple measures of 'screen time' or 'amount of social media use' through use of the Digital Activities and Feelings Inventory, which was co-designed with adolescents to identify and distinguish a range of digital activities, including risky activities, and the cognitive and affective reactions they evoke.
- Furthermore, the study will assess the impact of cognitive and affective responses to digital activities as a predictor of depression symptoms.
- Since the meaning and impact of digital activity are likely to vary across adolescence, the study focuses on mid-adolescence (13-14 years old at baseline), a period when the likelihood of depression rises.
- It will not be feasible to implement a reliable independent measure of adolescents' digital activity and mental health, although we will collect proxy measures from parents.

3 Introduction

Adolescence is a unique period of substantial biological, psychological and social changes. It is also a period that is associated with an increased likelihood of developing mental health problems,

For peer review only - http://bmjopen.bmj.com/site/about/guidelines.xhtml

especially depression (1, 2). In Western countries, adolescent mental health problems have been steadily increasing over the past three decades. In the UK, for adolescents aged 17 to 19, rates of probable mental disorders rose from *1 in 10* in 2017 to *1 in 4* in 2022 (3). In parallel, there has been a consistent increase in the ownership of digital devices (e.g., smartphones, laptops, game consoles). For example, in the UK, 98% of 12-15-year-old adolescents own a mobile phone (4). Inevitably, digital activity has become an integral part of adolescent life with many adolescents reporting to be almost constantly online (5, 6). This has led to concerns that engaging in digital activity may be harmful to adolescent mental health and psychological wellbeing (7).

However, research findings are nuanced (8), with small effect sizes (9), and subject to measurement problems (10) that require careful contextualisation (11). Systematic reviews of the literature on the link between digital activity and mental health reveal mixed and inconsistent results (7, 12-15). Specifically, results vary depending on the type of online risk (16) and users' mood and susceptibility (17), as well as on their vulnerability and available support (18). At the same time, the potential benefits of digital activity have been highlighted, including opportunities for social connection, support and entertainment (19, 20) and greater control over how one selects and engages with the external environment (21). Indeed, research identifies the benefits of digital activity, though few studies attempt or find links with robust measures of positive mental health or wellbeing (22, 23). Literature reviews often call for more specificity in the construction of measures and research designs (24, 25).

Finding adequate measures of digital activity has proved particularly challenging, with many studies using a simple measure of 'screen time' or 'social media use' (26). This is important because different digital activities might be linked to different mental health outcomes (27). That is, some digital activities are more likely to contribute to poor mental health than others. For example, seeing

For peer review only

For peer review only - http://bmjopen.bmj.com/site/about/guidelines.xhtml

violent or self-harm content, being treated in a hurtful way, or comparing oneself adversely to others are likely to confer risks for mental health and hereafter will be referred to as 'risky digital activities' (28, 29, 30). By contrast, entertainment activities, such as playing a digital game or having fun with other people online, are likely to be either neutral or perhaps even protective for mental health. Mental health outcome measures can also be problematic, with some studies failing to distinguish between different mental health conditions, even though the effects may differ for anxiety and depression (31) and the effects for wellbeing may not simply be the inverse of those for depression (32). Regarding research design, prior studies have often mixed participants of different ages or developmental stages, although the effects may be developmentally specific (33). However, the most criticised aspect of prior designs is reliance on cross-sectional data even though it is vital to clarify the causal direction: does digital activity increase the likelihood of developing mental health problems or does mental health determine digital activity (34)? Related to this issue of causality, is locating the effects of digital activity in the context of offline risk and protective factors. For example, controlling for confounding risks may change the digital activity-mental health relationship (35-37). Other potential mediating or moderating mechanisms may also drive or alter the relationship between digital activity and mental health (38).

4 Aims and objectives

To address these limitations, the present study, which is funded by the Medical Research Council as part of a large multi-institutional programme of research called Digital Youth https://digitalyouth.ac.uk (funder reference MR/W002450/1; Chief Investigators: Professors Hollis and Townsend, University of Nottingham), aims to examine the reciprocal longitudinal direct and indirect pathways between adolescent mental health and wellbeing and digital activity.

For peer review only - http://bmjopen.bmj.com/site/about/guidelines.xhtml

The study was designed to test a recently proposed cognitive-affective theory of the complex and reciprocal relationships between digital activity and depression (39). In this study, we propose three core hypotheses.

First, risky digital activity impacts on depression through the frequency and persistence of the negative reactions it evokes in individuals. These encompass both negative affect (e.g., anger, annoyance) and negative cognitions (e.g., "I am stupid", "I am inferior").

Second, depression will, in turn, exacerbate risky digital activities (i.e., leading individuals to engage in activities that contribute to poor mental health) or the amount of negative affective and cognitive reactions it evokes, creating the potential for destructive cycles that may alter the course of development.

Third, individuals who recognise the negative impact of digital activity on their mental health might be able to mitigate it by either reducing their risky digital activities and/or by modifying their reactions to it. This, in turn, will lead to better mental health outcomes.

The *primary objective* of this study is to examine whether digital activity predicts an increase in depression symptoms over a 12-month period in middle adolescence (i.e., age 13-14 years), the developmental period when depression likelihood is beginning to increase (40).

Secondary objectives are to examine:

1. Whether depression is prospectively associated with risky digital activities over a 12-month period.

For peer review only - http://bmjopen.bmj.com/site/about/guidelines.xhtml

2. Whether the pathway from digital activity to depression is mediated by affective and cognitive reactions evoked by digital activity.

3. Whether, reciprocally, depression feeds back to alter patterns of digital activity and the reactions it evokes.

4. Whether personal resilience and other known risk and protective factors moderate the pathway from digital activity to depression and vice versa.

5. Whether effects observed for wellbeing are the inverse of those observed for depression.

6. Explore whether an individual's reflections about the impact their digital activity is having on their mental health leads to their modifying their pattern of digital activity and/or their reactions to it.

We will also explore which factors predict high-risk digital activities and what proportion of adolescents are concerned about the impact of digital activity on their lives and mental health. What proportion attempts to modify their activity in light of this? What approaches do they use?

5 Methods and analysis

5.1 Study design

This is a longitudinal observational study with measures taken at baseline (T1), 6 months (T3), and 12 months (T3) post-baseline.

5.2 Study setting

All study procedures (consent form and questionnaire completion) will be completed remotely using Qualtrics. Participants will either complete the study measures at school or use their own devices connected to the internet in their preferred setting.

For peer review only - http://bmjopen.bmj.com/site/about/guidelines.xhtml

5.3 Participant selection and eligibility criteria

Participants in the study will be adolescents and their parents. Adolescents will be recruited from secondary schools in the United Kingdom. Each adolescent will nominate a parent (or caregiver/guardian), who will also be invited to participate. To ensure participants represent a wide socioeconomic spectrum, we will approach a range of schools including mainstream and alternative provision schools, either state-funded or independent. To identify schools, we will use contacts in our professional networks as well as information from the Department for Education https://get-information-schools.service.gov.uk. We will continue approaching schools with a request to support recruitment until sufficient adolescents have consented to the study. A member of the research team will meet with each school's nominated representative to discuss the study.

5.3.1 Inclusion criteria

- Adolescents attending a secondary school in England, Wales or Scotland.
- The adolescents are aged 13-14 years old at the time of enrolment (typically, Year 9 in England and Wales or S3 in Scotland).
- Adolescents with access to a digital device connected to the internet (e.g., owned or shared at home, or a publicly shared device).
- There are no specific inclusion criteria for parents.

5.3.2 Exclusion criteria

- Adolescents will be excluded if they are unable to complete assessments independently.

5.4 Sample size justification

We judged the minimum clinically meaningful effect size of interest to detect, based on potential public health significance, to be r = .2. Our cross-sectional pilot study (publication forthcoming)

For peer review only - http://bmjopen.bmj.com/site/about/guidelines.xhtml

found correlations with much larger effect sizes than this between proposed predictors and outcomes – suggesting that this effect size is obtainable. An *a priori* biserial normal model was used to calculate the required sample size (41). To achieve 80% power for an alpha of .05, this power analysis indicated a sample size of 193 is needed. Assuming a conservative 70% retention at 12-month follow-up, we will recruit a minimum of n = 276 adolescents; however, we will aim for the sample to be larger to allow sufficient power for moderation/mediation analyses.

5.5 Recruitment and follow-up procedures

Researchers will work with schools to inform adolescents' and parents' about the study and to provide them with opportunities to ask questions. This may involve visiting schools to talk about the study during assemblies/classes, asking schools to display posters and share flyers with eligible students, and circulating study information via the school's newsletter, website, and social media. Schools will also be asked to share a digital recruitment pack with each adolescent, which will include study promotion materials (e.g., a flyer) and information sheets for adolescents and parents. The information sheet will include a link (e.g., a QR code) to an online consent form for the adolescent.

The school will also choose one of the two approaches regarding parental consent, that is, the opt-in and opt-out options. Under the opt-in approach, adolescents will not be able to participate unless the parent (or legal guardian) actively consents to their child's participation by filling in an online consent form. Under the opt-out approach, adolescents will be asked to seek verbal consent from their parent (or legal guardian) and will be able to participate unless their parent (or legal guardian) explicitly chooses for their child not to participate in the study. Under both consent approaches, adolescents and parents will need to consent to their own participation.

For peer review only

Adolescents and their parents will provide informed consent online. Following consent, adolescents and parents will receive a notification via email and/or text message to complete the battery of T1 questionnaires online. Each assessment window will close after three weeks of opening. A notification to complete assessments will be sent via email and/or text message on the day that the assessment is due to be completed.

Each adolescent will receive a £15 voucher for completing the questionnaires at each time point and a 'bonus' of £15 for completing assessments at all timepoints (a total of £60 for those who complete the whole study). This compensation structure was suggested by the DIORA youth panel as the most optimal to maintain adolescents' engagement in the study. Parents will be entered into a prize draw. Each school facilitating recruitment will receive between £100 and £250 to thank them for their support (amount depending on the level of support given to the study's recruitment). Other non-monetary incentives (e.g., psychology talks, school-level feedback, etc.) will be negotiated with individual schools.

5.6 Participant timeline

Adolescent and parent measures will be completed at T1, T2, and T3. A detailed schedule of assessments is presented in Supplementary Table 1.

5.7 Outcomes

5.7.1 Adolescent-reported outcomes

Adolescents will report their depression and anxiety symptoms using the *Revised Child Anxiety and Depression Scale 25–Youth Version* (RCADS-25; 42). Adolescents will rate how often each statement applies to them on a four-point scale (0 = never, 3 = always). The subscale score is derived by summing individual item scores. Higher scores on the subscale indicate greater symptoms of depression and/or anxiety. Conduct problems and attention-deficit hyperactivity symptoms will be

For peer review only - http://bmjopen.bmj.com/site/about/guidelines.xhtml

For peer review only

measured with the respective subscales of The *Strengths and Difficulties Questionnaire* (SDQ; 43). In this 25-item questionnaire adolescents will be provided with descriptions of personal characteristics and asked to indicate whether the description is true of themselves on a 3-point rating (0 = "not true" to 2 = "certainly true"). After reverse coding, individual item scores are summed to derive an overall subscale score where the higher score represents greater severity of symptoms. Wellbeing will be measured with the *Warwick-Edinburgh Mental Wellbeing Scale* (WEMWBS; 44). It assesses the presence of positive thoughts and feelings, which relate to one's general functioning. All statements are positively worded and are rated on a 5-point rating scale (1 = "none of the time" to 5 = "all of the time"). Individual item scores are summed to calculate the total score.

Screen time will be measured with a single question: "On an ordinary school day, about how long do you send on your phone or the internet (not counting time for schoolwork)?" Responses are provided on a 9-point scale ("little to no time" to "about 7 hours"). Adolescents will also complete a detailed assessment of their digital activity using the recently developed *My Online Activity* subscale of the *Digital Activity and Feelings Inventory* (DAFI). It measures four distinct dimensions of digital activity: high-risk content (7 items; e.g., "I watched some sad or dark content"), high-risk conduct (8 items; e.g., "I saw people talk about or show ways of being very thin"), entertainment (3 items; e.g. "I played games with others"), and social use (4 items; e.g., "I like or shared other people's posts"). Adolescents will rate how often they did or experienced each item in the past two weeks on a 5-point scale (0 = "never" to 4 = "at least every day"). Reactions evoked by digital activity will be measured with the *My Feelings Online* subscale of the DAFI, which includes 10 positive and 10 negative feelings that encompass both general emotional reactions (e.g., "stressed", "calm") and those that specifically pertain to the self (e.g., "insecure", "attractive", "loved"). Adolescents will rate how often they experienced each item in relation to being online in the past two weeks on a 5-point scale (0 =

For peer review only - http://bmjopen.bmj.com/site/about/guidelines.xhtml

“never” to 4 = “at least every day”). Information about the validation of this measure will be reported in the forthcoming publication The impact of online activities on daily life and management behaviours will be measured by a second new measure: the *My Life Online* (MYLO) questionnaire. Section one (9 items), asks adolescents about how much their life was impacted by their digital activity (e.g., “I missed meals”, “I had difficulties at school/college/university”) rated on a scale of 0 = “never” to 4 = “at least every day” and whether they were concerned about this (e.g., “I worried how being online affected my mental health”) rated on a scale of 0 = “not at all” to 4 = “very much”. In section two of MYLO adolescents (14 items) will rate the extent they tried to reduce the negative effects of their digital activity (e.g., “Took a break from social media”, “Removed or blocked accounts”). Responses are provided on a scale ranging from 0 = “never” to 4 = “at least every day”.

Finally, we will measure the following individual characteristics*:* psychological resilience, social comparison, self-efficacy, emotion regulation and social desirability. We will also measure the following contextual factors: offline events and experiences, loneliness, social support and the presence of significant life events. A demographic questionnaire will ask for information about adolescents’ age, biological sex and gender identity, ethnicity, eligibility for free school meals, and perceived family affluence. A detailed description of al the scales and their administration is provided in Supplementary Table 1 (see the supplementary materials).

5.7.2 Parent-reported outcomes

Parents will report their child’s depression and anxiety symptoms on the respective subscales of the RCADS-25. Adolescent psychological wellbeing will be measured with an adapted version of the WEMWBS. Parents will also complete a parent-report version of MYLO to rate how much being online has affected their child’s everyday life and will rate perceived positive and negative impacts of their child’s online activity on their mental health and relationships. They will also report on their

For peer review only - http://bmjopen.bmj.com/site/about/guidelines.xhtml

relationship with their child on the Child-Parent Relationship Scale – Short Form (45) which includes two subscales: the 7-item closeness subscale scale and the 8-item conflict subscale. Parents will rate how much each statement applies to their relationship with their child on a 5-point rating scale ranging from “definitely does not apply” = 1 to “definitely applies” = 5.

Parental depression will be measured with The Patient Health Questionnaire (46)which assesses depression symptoms using a 4-point scale ranging from “not at all” = 0 to “nearly every day” = 3. Individual item scores are summed to derive an overall score – a higher overall score represents more severe depression symptoms. Parental wellbeing will be measured with the WEMWBS.

To obtain demographic information, parents will provide information about their own ethnicity, education and employment status, as well as report the occupation of the main earner in the family. The latter will be categorised using the nine major groups from the Office for National Statistics Standard Occupational Classification Hierarchy (47).

5.8 General procedure

5.8.1 Data collection methods

Data will be collected online using Qualtrics – a secure dedicated survey platform. Each consenting participant (i.e., adolescent and parent) will receive a link to the relevant T1 questionnaires. If a participant does not start the T1 questionnaires within three weeks, it will be assumed they do not wish to continue and will not be invited to complete follow-up assessments.

Participants who completed T1 questionnaires will be sent two follow-up assessment invitations (T2 and T3) via text message or email. At each time point, they will have three weeks to start completing the questionnaires and seven days to finish the questionnaires once started. Up to three reminders will be sent to those who have not completed the assessment.

For peer review only - http://bmjopen.bmj.com/site/about/guidelines.xhtml

5.8.2 Withdrawals

Participants will be free to withdraw at any point in the study by either ignoring invitations to complete the online questionnaires or by emailing the research team. Data collected before withdrawal will be included in the analysis.

5.9 Public patient involvement and engagement: youth panel

The overall Digital Youth programme research priorities, ethical and design considerations were considered at the pre-funding stage through the consultation with the MRC Adolescence, Mental Health & Developing Mind Engagement Award Young Person Advisory Group (now Sprouting Minds), a youth panel which informs and supports Digital Youth researchers through co-facilitation and collaboration.

In addition, DIORA has a dedicated youth advisory panel comprising adolescents aged 13-17, which meets online approximately every two months. The DIORA youth panel played a crucial role in the development of the DAFI to ensure that the list of digital activities and reactions they induce were relevant to adolescents. The panel also reviewed and advised on current study plans and procedures and participant-facing documents to ensure these are acceptable and inclusive. For example, the panel helped to short-list digital activities that are relevant to contemporary adolescents and advised that recruitment occurs through schools to increase the perceived legitimacy of research (also because students may not trust or know the academic institutions that are conducting the study). We will continue working with both Sprouting Minds and the DIORA youth panel throughout this project and future activities will include reviewing the progress of the longitudinal study, interpreting findings, and involvement in results dissemination.

For peer review only - http://bmjopen.bmj.com/site/about/guidelines.xhtml

5.10 Analysis plan

We will examine information about the proportion of missing data and its patterns. Summary statistics will be used to describe demographic information and provide information about the level of psychopathology symptoms and digital activity in the adolescent sample.

We will fit a range of models to examine the associations between digital activity, depression and wellbeing drawing on the structural equation modelling framework, which provides the flexibility to implement a diversity of models which map to the study's objectives. For example, (random-intercept) cross-lagged panel models will be used to examine (potentially reciprocal) directional relations over time between digital activity and depression; growth curve models will be used to examine trajectories over time, their individual differences, and predictors of these individual differences; and mediating paths can be added to models to examine indirect effects or 'mechanistic pathways'. Missing data will be dealt with using methods such as multiple imputation, full information maximum likelihood estimation, and Bayesian estimation but the specific approach will depend on the model, reflecting the greater compatibility of some approaches with certain modelling and estimation frameworks. Further details of the analyses will be specified in the statistical analysis plan.

6 Ethics and dissemination

6.1 Ethics approval and safety consideration

The study was approved by the London School of Economics Research Ethics Committee, reference number 249287. Any future protocol amendments will be documented and submitted for ethical approval prior to implementation.

For peer review only - http://bmjopen.bmj.com/site/about/guidelines.xhtml
For peer review only

Given the observational design, the risk of harm to participants is minimal. There is a small likelihood that completing study questionnaires may result in new concerns related to parent or child mental health, digital activity, relationships, or affective and cognitive reactions. This risk is clearly explained through the information sheet. All questionnaires/procedures have been reviewed by the DIORA youth panel to ensure sensitivity and acceptability to adolescents and their parents.

All participants will give written (electronic) informed consent for their participation. For adolescents recruited via the opt-in approach, we will also seek written parental informed consent (for details see 5.5. ‘Recruitment and follow-up procedures’). Participants will be able to skip any question they do not want to answer, and all participants will be provided with information on seeking mental health support and contacts for national crisis services at the start and end of each assessment battery.

6.2 Dissemination

Results will be published in a peer-reviewed scientific journal and included in the funder report. Authorship will be determined according to the Digital Youth’s publication policy developed on the basis of the Committee on Publication Ethics (COPE) standards based on individual contributions. Findings will be disseminated through presentations at conferences and seminars in the UK and overseas. There will also be a general dissemination through the Digital Youth and institutional websites, social media, and stakeholder engagement activities.

6.3 Study oversight

Overall programme operational oversight and progress monitoring is provided by the Digital Youth leadership team. This group comprises the programme Principal Investigators (Profs. Hollis and Townsend), work package leads, Programme Manager and members of Sprouting Minds. Overall scientific oversight is provided by the Scientific Advisory Board which reviews and approves study

For peer review only - http://bmjopen.bmj.com/site/about/guidelines.xhtml

protocols and receives progress updates. This comprises international academic experts representing disciplines relevant to the programme (e.g., adolescent mental health, computer science, digital psychological interventions).

7 Study status

The study recruitment started in October 2023 with data collection planned to be completed in March 2025.

8 Funding

The authors (MS, KKA, JB, EA, CH, ET, SL and ESB) acknowledge the support of the UK Research and Innovation (UKRI) Digital Youth Programme award [MRC project reference MR/W002450/1] which is part of the AHRC/ESRC/MRC Adolescence, Mental Health and the Developing Mind programme. The project funding covers all costs in relation to the study, including but not limited to, higher education institutions' costs, equipment, and the youth panel costs. The funding covers the period from 1 September 2021 to 31 August 2025.

9 Author contributions

CH and ET are the overall Digital Youth programme chief investigators. The guarantors of the study are Sonia Livingstone and Edmund Sonuga-Barke (SL and ESB); they accept full responsibility for the conduct of the study, will have access to the data, and will control the decision to publish. SL, ESB, MS, JB, AM and KKA contributed to the design of the study. AJ, ESB and SL contributed to the analysis plans. MS and JB prepared the protocol and other study documentation for ethical approval. KKA and MS prepared the first draft of the manuscript for publication. EA, JB and NN organised the activities of the DIORA youth panel and liaised with Sprouting Minds. All authors

For peer review only

For peer review only - http://bmjopen.bmj.com/site/about/guidelines.xhtml

contributed to drafting and revising the manuscript and approved the final version to be submitted for publication.

10 Competing interests

There are no competing interests for any of the authors.

11 References

1. Weavers B, Riglin L, Martin J, Anney R, Collishaw S, Heron J, et al. Characterising depression trajectories in young people at high familial risk of depression. Journal of Affective Disorders. 2023;337:66-74.
2. Wiederhold BK. The escalating crisis in adolescent mental health. Mary Ann Liebert, Inc., publishers 140 Huguenot Street, 3rd Floor New …; 2022. p. 81-2.
3. Newlove-Delgado T, Marcheselli F, Williams T, Mandalia D, Davis J, McManus S, et al. Mental Health of Children and Young People in England, 2022-wave 3 follow up to the 2017 survey. 2022.
4. Ofcom. Children and parents: media use and attitudes report 2023. 2023 29 March 2023.
5. Nagata JM, Cortez CA, Cattle CJ, Ganson KT, Iyer P, Bibbins-Domingo K, et al. Screen time use among US adolescents during the COVID-19 pandemic: findings from the adolescent brain cognitive development (ABCD) study. JAMA pediatrics. 2022;176(1):94-6.
6. Thomas G, Bennie JA, De Cocker K, Castro O, Biddle SJ. A descriptive epidemiology of screen-based devices by children and adolescents: a scoping review of 130 surveillance studies since 2000. Child Indicators Research. 2020;13:935-50.
7. Odgers CL, Jensen MR. Annual research review: Adolescent mental health in the digital age: Facts, fears, and future directions. Journal of Child Psychology and Psychiatry. 2020;61(3):336-48.
8. Ferguson CJ. Does the internet make the world worse? Depression, aggression and polarization in the social media age. Bulletin of Science, Technology & Society. 2021;41(4):116-35.
9. Steinsbekk S, Nesi J, Wichstrøm L. Social media behaviors and symptoms of anxiety and depression. A four-wave cohort study from age 10–16 years. Computers in Human Behavior. 2023;147:107859.
10. Parry DA, Davidson BI, Sewall CJ, Fisher JT, Mieczkowski H, Quintana DS. A systematic review and meta-analysis of discrepancies between logged and self-reported digital media use. Nature Human Behaviour. 2021;5(11):1535-47.
11. Panayiotou M, Black L, Carmichael-Murphy P, Qualter P, Humphrey N. Time spent on social media among the least influential factors in adolescent mental health: preliminary results from a panel network analysis. Nature Mental Health. 2023;1(5):316-26.
12. Orben A. Teenagers, screens and social media: a narrative review of reviews and key studies. Social Psychiatry and Psychiatric Epidemiology. 2020;55(4):407-14.
13. Keles B, McCrae N, Grealish A. A systematic review: the influence of social media on depression, anxiety and psychological distress in adolescents. International journal of adolescence and youth. 2020;25(1):79-93.
14. Kostyrka-Allchorne K, Stoilova M, Bourgaize J, Rahali M, Livingstone S, Sonuga-Barke E. Digital experiences and their impact on the lives of adolescents with pre-existing anxiety, depression, eating and nonsuicidal self-injury conditions–a systematic review. Child and Adolescent Mental Health. 2023;28(1):22-32.
15. Sanders T, Noetel M, Parker P, Del Pozo Cruz B, Biddle S, Ronto R, et al. An umbrella review of the benefits and risks associated with youths' interactions with electronic screens. Nature Human Behaviour. 2023.
16. Livingstone, S., Stoilova, M., & Rahali, R. The 4Cs: classifying online risk to children. CORE Short Report Series on Key Topics. 2021. Hamburg: Leibniz-Institut für Medienforschung | Hans-Bredow-Institut (HBI). DOI: 10.21241/ssoar.71817
17. Valkenburg PM, van Driel II, Beyens I. The associations of active and passive social media use with well-being: A critical scoping review. New media & society. 2022;24(2):530-49.

For peer review only - http://bmjopen.bmj.com/site/about/guidelines.xhtml

18. El-Asam A, Lane R, Katz A, editors. Psychological distress and its mediating effect on experiences of online risk: The case for vulnerable young people. Frontiers in Education; 2022: Frontiers.
19. Hollis C, Livingstone S, Sonuga-Barke E. The role of digital technology in children and young people's mental health–a triple-edged sword? : Wiley Online Library; 2020. p. 837-41.
20. Anthony R, Young H, Hewitt G, Sloan L, Moore G, Murphy S, et al. Young people's online communication and its association with mental well-being: results from the 2019 student health and well-being survey. Child and Adolescent Mental Health. 2023;28(1):4-11.
21. Sonuga-Barke E, Kostyrka-Allchorne K. Editorial Perspective: Attention-deficit/hyperactivity disorder viewed as neuro-divergence in the digital world. Journal of Child Psychology and Psychiatry. 2023;64(6):972-4.
22. O'Reilly M. Social media and adolescent mental health: the good, the bad and the ugly. Journal of Mental Health. 2020;29(2):200-6.
23. Halbrook YJ, O'Donnell AT, Msetfi RM. When and how video games can be good: A review of the positive effects of video games on well-being. Perspectives on Psychological Science. 2019;14(6):1096-104.
24. Badu, E., O'Brien, A.P. & Mitchell, R. An integrative review on methodological considerations in mental health research – design, sampling, data collection procedure and quality assurance. Arch Public Health. 2019; 77(37):1-15.
25. Breedvelt J, Zamperoni V, South E, Uphoff EP, Gilbody S, Bockting CLH, Churchill R, Kousoulis, AA. A systematic review of mental health measurement scales for evaluating the effects of mental health prevention interventions. European Journal of Public Health. 2020; 30(3): 510–516.
26 Browne DT, May SS, Colucci L, Hurst-Della Pietra P, Christakis D, Asamoah T, et al. From screen time to the digital level of analysis: a scoping review of measures for digital media use in children and adolescents. BMJ open. 2021;11(5):e046367.
27. Tang S, Werner-Seidler A, Torok M, Mackinnon AJ, Christensen H. The relationship between screen time and mental health in young people: A systematic review of longitudinal studies. Clinical psychology review. 2021;86:102021.
28. Sadza A, Rozendaal E, Daalmans S, Buijzen M. Let's talk about risks. Parental and peer mediation and their relation to adolescents' perceptions of on- and off-screen risk behavior. Communications. 2024; 49(2): 175-198.
29. Godard R, Holtzman S. Are active and passive social media use related to mental health, wellbeing, and social support outcomes? A meta-analysis of 141 studies. Journal of Computer-Mediated Communication. 2024; 29(1): zmad055.
30. Murray A, Wadman R, Townsend E. Do young people who self-harm experience cognitions and emotions related to post-traumatic growth? Journal of Affective Disorders Reports. 2024; 15: 100683.
31. Kim S, Favotto L, Halladay J, Wang L, Boyle MH, Georgiades K. Differential associations between passive and active forms of screen time and adolescent mood and anxiety disorders. Social psychiatry and psychiatric epidemiology. 2020;55:1469-78.
32. Stiglic N, Viner RM. Effects of screentime on the health and well-being of children and adolescents: a systematic review of reviews. BMJ open. 2019;9(1):e023191.
33. El Asam A, Samara M, Terry P. Problematic internet use and mental health among British children and adolescents. Addictive behaviors. 2019;90:428-36.
34. Boer M, Stevens GW, Finkenauer C, de Looze ME, van den Eijnden RJ. Social media use intensity, social media use problems, and mental health among adolescents: Investigating directionality and mediating processes. Computers in Human Behavior. 2021;116:106645.
35. Jensen M, George MJ, Russell MR, Odgers CL. Young adolescents' digital technology use and mental health symptoms: Little evidence of longitudinal or daily linkages. Clinical Psychological Science. 2019;7(6):1416-33.
36. Schemer C, Masur PK, Geiß S, Müller P, Schäfer S. The impact of internet and social media use on well-being: A longitudinal analysis of adolescents across nine years. Journal of Computer-Mediated Communication. 2021;26(1):1-21.
37. Paulich KN, Ross JM, Lessem JM, Hewitt JK. Screen time and early adolescent mental health, academic, and social outcomes in 9- and 10- year old children: Utilizing the Adolescent Brain Cognitive Development ℠ (ABCD) Study. PLoS One. 2021;16(9):e0256591.
38. Granic I, Morita H, Scholten H. Beyond screen time: Identity development in the digital age. Psychological Inquiry. 2020;31(3):195-223.

For peer review only - http://bmjopen.bmj.com/site/about/guidelines.xhtml

39. Sonuga-Barke E, Stoilova M, Kostyrka-Allchorne K, Bourgaize J, Hollis C, Townsend E, et al. Pathways between digital activity and depressed mood in adolescence: outlining a developmental model integrating risk, reactivity, resilience and reciprocity. 2024; 58:101411.
40. Grossberg A, Rice T. Depression and suicidal behavior in adolescents. Medical Clinics. 2023;107(1):169-82.
41. Faul F, Erdfelder E, Buchner A, Lang A-G. Statistical power analyses using G* Power 3.1: Tests for correlation and regression analyses. Behavior research methods. 2009;41(4):1149-60.
42. Ebesutani C, Korathu-Larson P, Nakamura BJ, Higa-McMillan C, Chorpita B. The revised child anxiety and depression scale 25–parent version: scale development and validation in a school-based and clinical sample. Assessment. 2017;24(6):712-28.
43. Goodman R. The Strengths and Difficulties Questionnaire: a research note. Journal of child psychology and psychiatry. 1997;38(5):581-6.
44. Tennant R, Hiller L, Fishwick R, Platt S, Joseph S, Weich S, et al. The Warwick-Edinburgh Mental Well-being Scale (WEMWBS): development and UK validation. Health and Quality of Life Outcomes. 2007;5(1):63.
45. Driscoll K, Pianta RC. Mothers' and Fathers' Perceptions of Conflict and Closeness in Parent-Child Relationships during Early Childhood. Journal of Early Childhood & Infant Psychology. 2011(7).
46. Kroenke K, Spitzer RL, Williams JB. The PHQ-9: validity of a brief depression severity measure. J Gen Intern Med. 2001;16(9):606-13.
47. ONS. 2023 [Available from: https://onsdigital.github.io/dp-classification-tools/standard-occupational-classification/ONS_SOC_hierarchy_view.html]

For peer review only

For peer review only - http://bmjopen.bmj.com/site/about/guidelines.xhtml

Table 1. DIORA constructs, outcome, measures and schedule of assessments.

General construct	Outcome	Measure	T1	T2	T3
Demographic information		Adolescent age, biological sex, gender identity, ethnicity	A, P		
		Family affluence, eligibility for free school meals	A		
		Parental ethnicity, parental education, and employment status, main earner occupation.	P		
Adolescent psychopathology	Depression symptoms (primary outcome at T3)	Revised Child Anxiety and Depression Scale 25–Youth Version (RCADS-25; Ebesutani et al., 2017)	A, P	A, P	A, P
	Anxiety symptoms	Anxiety subscale of the RCADS-25.	A, P	A, P	A, P
	ADHD symptoms and conduct problems	Respective subscales of The Strengths and Difficulties Questionnaire (SDQ; Goodman, 1997).	A		A
Adolescent wellbeing	Wellbeing	Warwick-Edinburgh Mental Wellbeing Scale (WEMWBS; Tennant et al., 2007)	A, P	A, P	A, P
Digital activity and screen time	Amount of time spent online	Screen time measure	A	A	A
	Digital activity	*My Online Activity* subscale of the Digital Activity and Feelings Inventory (DAFI)	A	A	A
	Emotional reactions induced by digital activity	*My Feelings Online* subscale of the DAFI	A	A	A
	Impact of digital activity on daily functioning and concerns about the impact of digital activity on mental health and relationships	**My Life Online** (MYLO) questionnaire. Section one (9 items), asks adolescents about how much their life was impacted by their digital activity (e.g., "I missed meals", "I had difficulties at school/college/university") rated on a scale of 0 = "never" to 4 = "at least every day" and whether they were concerned about this (e.g., "I worried how being online affected my mental health") rated on a scale of 0 = "not at all" to 4 = "very much".<br><br>Parents will complete the parent version of **My Life Online** (MYLO). Parents will rate how much being online has affected their child's everyday life in the past two weeks (e.g., "My child missed meals", "My child had difficulties at school"). Responses will be provided on a scale ranging from "never" = 0 to "at least every day" = 4. They will also rate perceived positive and negative impacts of their child's online activity on their mental health and	A, P	A, P	A, P

For peer review only - http://bmjopen.bmj.com/site/about/guidelines.xhtml

		relationships (e.g., "Being online positively affected my child's mental health" or "My child's online activity damaged our relationship"). Responses will be provided on a 5-point scale ranging from "not at all" = 0 to "very much" = 4. After reverse coding responses to items regarding positive impacts, item scores will be summed to derive an overall negative impact score.			
	Self-management of digital activity	In section two of MYLO adolescents (14 items) will rate the extent they tried to reduce the negative effects of their digital activity (e.g., "Took a break from social media", "Removed or blocked accounts"). Responses are provided on a scale ranging from 0 = "never" to 4 = "at least every day".	A	A	A
Individual characteristics	Social comparison	**Social Comparison Scale** (SCS; Allan & Gilbert, 1995). Adolescents will rate how they compare themselves to others using 10 pairs of opposite characteristics (e.g., "inferior-superior", "weaker-stronger") on a scale ranging from 1 to 10. Individual item scores are summed to derive an overall score where a higher score represents a more favourable comparison of oneself in respect to others.	A	A	A
	Social desirability	The **Children's Social Desirability – Short Scale** (CSD-SS; Miller et al., 2014). Adolescents will answer "yes" or "no" to each of 14 items and a higher score represents a greater tendency to answer in a more socially desirable manner.	A		
	Self-efficacy	The 10-item **Generalised Self-Efficacy Scale** (GSES; Schwarzer & Jerusalem, 1995), which measures one's perceptions of their ability to perform novel or difficult tasks, or cope with adversity. Responses are provided on a 4-point scale ranging from 1 = "not at all true" to 4 = "exactly true" and are summed to derive an overall score.	A	A	A
	Resilience	The **Youth Online and Offline Resilience Scale** (YOORS; adapted from Smith et al., 2008). This 8-item measure includes the original 6 items of the Brief Resilience Scale measuring domain-general resilience and two new items relating specifically to resilience online (e.g., "When something upsets me online, I tend to get over it quickly"). Responses are made on a 5-point scale ranging from 1 = "strongly disagree" to 5 = "strongly agree".	A	A	A
	Emotion regulation	**Emotion Regulation Questionnaire for Children and Adolescents** (ERQ-CA; Gullone & Taffe, 2012). The ERQ-CA measures two regulation strategies with the respective subscale scores. Cognitive reappraisal subscale (6 items) measures the extent to which an individual actively attempts to change how they think about a situation to change its emotional impact. Expressive suppression subscale (4 items) measures the extent to which an individual inhibits the behavioural expression of their emotions (Gross & John, 2012). Responses are made on a 7-point scale, ranging from 1 = "strongly disagree" to 7 = "strongly agree" and summed for the respective subscales. Higher scores indicated higher usage of the strategy.	A	A	A

For peer review only

Contextual factors	Social support	Social support will be measured with the **Social Support Questionnaire - 6** (SCQ; Sarason et al., 1987). This 12-item questionnaire measures availability and satisfaction with support. To measure the availability of social support, adolescents will state how many people (ranging from "0" to "7 and more") they can count on to receive various forms of support. Then they rate how satisfied they are with the support they receive using a 6-point rating scale ranging from "very dissatisfied" to "very satisfied".	A	A	A
	Loneliness	Loneliness will be measured with the 4-item **UCLA Loneliness Questionnaire** (Russell et al., 1980). Responses are made on 4-point scale ranging from 0 = 'never' to 3 = "often' and individual item scores are summed to derive an overall loneliness score.	A	A	A
	Offline experiences and events	Frequency of offline experiences will be measured with the newly developed **Personal Experiences in Everyday Life** (PEEL) questionnaire. This is a new 19-item measure consisting of positive (e.g., hobbies, exercise) and negative (e.g., drinking alcohol, missing school) offline experiences and activities. Responses are provided on a 5-point scale ranging from 0 = "never" to 4 = "at least every day".	A	A	A
	Substantial life events	The presence of significant life events in adolescence (e.g., moving home, parental job loss, new sibling) will be measured with a 10-item questionnaire adapted from the **Adverse Life Events Checklist** (Tiet et al., 2001). Adolescents will respond "yes' = 1 or "no" = 0 and responses will be summed to derive an overall score.	A	A	A
Parental mental health and wellbeing	Depression symptoms	**The Patient Health Questionnaire** (PHQ-9; Kroenke et al., 2001). The PHQ-9 assesses depression symptoms using a 4-point scale ranging from "not at all" = 0 to "nearly every day" = 3. Individual items scores are summed to derive an overall score - higher overall score represents more severe depression symptoms.	P	P	P
	Wellbeing	Wellbeing will be measured with the WEMWBS.	P	P	P
Other parental measures	Child-parent relationship	The **Child-Parent Relationship Scale – Short Form** (CPRS-SF; Driscoll & Pianta, 2011), which includes two subscales: the 7-item closeness subscale scale and the 8-item conflict subscale. Parents will rate how much each statement applies to their relationship with their child on a 5-point rating scale ranging from "definitely does not apply" = 1 to "definitely applies" = 5.	P	P	P

For peer review only - http://bmjopen.bmj.com/site/about/guidelines.xhtml

STROBE Statement—Checklist of items that should be included in reports of ***cross-sectional studies***

	Item No	Recommendation	Page No
**Title and abstract**	1	(*a*) Indicate the study's design with a commonly used term in the title or the abstract	1-3
		(*b*) Provide in the abstract an informative and balanced summary of what was done and what was found	N/A
**Introduction**			
Background/rationale	2	Explain the scientific background and rationale for the investigation being reported	3-5
Objectives	3	State specific objectives, including any prespecified hypotheses	5-7
**Methods**			
Study design	4	Present key elements of study design early in the paper	7
Setting	5	Describe the setting, locations, and relevant dates, including periods of recruitment, exposure, follow-up, and data collection	7
Participants	6	(*a*) Give the eligibility criteria, and the sources and methods of selection of participants	8-9
Variables	7	Clearly define all outcomes, exposures, predictors, potential confounders, and effect modifiers. Give diagnostic criteria, if applicable	10-13
Data sources/ measurement	8*	For each variable of interest, give sources of data and details of methods of assessment (measurement). Describe comparability of assessment methods if there is more than one group	10-13 & *supplementary Table 1*
Bias	9	Describe any efforts to address potential sources of bias	N/A
Study size	10	Explain how the study size was arrived at	8-9
Quantitative variables	11	Explain how quantitative variables were handled in the analyses. If applicable, describe which groupings were chosen and why	N/A
Statistical methods	12	(*a*) Describe all statistical methods, including those used to control for confounding	15
		(*b*) Describe any methods used to examine subgroups and interactions	N/A
		(*c*) Explain how missing data were addressed	15
		(*d*) If applicable, describe analytical methods taking account of sampling strategy	N/A
		(*e*) Describe any sensitivity analyses	N/A
**Results**			
Participants	13*	(a) Report numbers of individuals at each stage of study—eg numbers potentially eligible, examined for eligibility, confirmed eligible, included in the study, completing follow-up, and analysed	N/A
		(b) Give reasons for non-participation at each stage	N/A
		(c) Consider use of a flow diagram	
Descriptive data	14*	(a) Give characteristics of study participants (eg demographic, clinical, social) and information on exposures and potential confounders	N/A
		(b) Indicate number of participants with missing data for each variable of interest	N/A

For peer review only

Outcome data	15*	Report numbers of outcome events or summary measures	N/A
Main results	16	(*a*) Give unadjusted estimates and, if applicable, confounder-adjusted estimates and their precision (eg, 95% confidence interval). Make clear which confounders were adjusted for and why they were included	N/A
		(*b*) Report category boundaries when continuous variables were categorized	N/A
		(*c*) If relevant, consider translating estimates of relative risk into absolute risk for a meaningful time period	N/A
Other analyses	17	Report other analyses done—eg analyses of subgroups and interactions, and sensitivity analyses	N/A
**Discussion**			
Key results	18	Summarise key results with reference to study objectives	N/A
Limitations	19	Discuss limitations of the study, taking into account sources of potential bias or imprecision. Discuss both direction and magnitude of any potential bias	N/A
Interpretation	20	Give a cautious overall interpretation of results considering objectives, limitations, multiplicity of analyses, results from similar studies, and other relevant evidence	N/A
Generalisability	21	Discuss the generalisability (external validity) of the study results	N/A
**Other information**			
Funding	22	Give the source of funding and the role of the funders for the present study and, if applicable, for the original study on which the present article is based	17

*Give information separately for exposed and unexposed groups.

Note: An Explanation and Elaboration article discusses each checklist item and gives methodological background and published examples of transparent reporting. The STROBE checklist is best used in conjunction with this article (freely available on the Web sites of PLoS Medicine at http://www.plosmedicine.org/, Annals of Internal Medicine at http://www.annals.org/, and Epidemiology at http://www.epidem.com/). Information on the STROBE Initiative is available at www.strobe-statement.org.

For peer review only
